# Supplementary material for: Purification and Characterization of a Novel Antifungal Flagellin Protein from Endophyte Bacillus methylotrophicus NJ13 against Ilyonectria robusta
Source: Microorganisms. 2019 Nov 22;7(12):605. doi: 10.3390/microorganisms7120605 (PMC6956119; doi:10.3390/microorganisms7120605)
Supplement: Supplementary file 1 [file microorganisms-07-00605-s001.pdf]

Appendix B

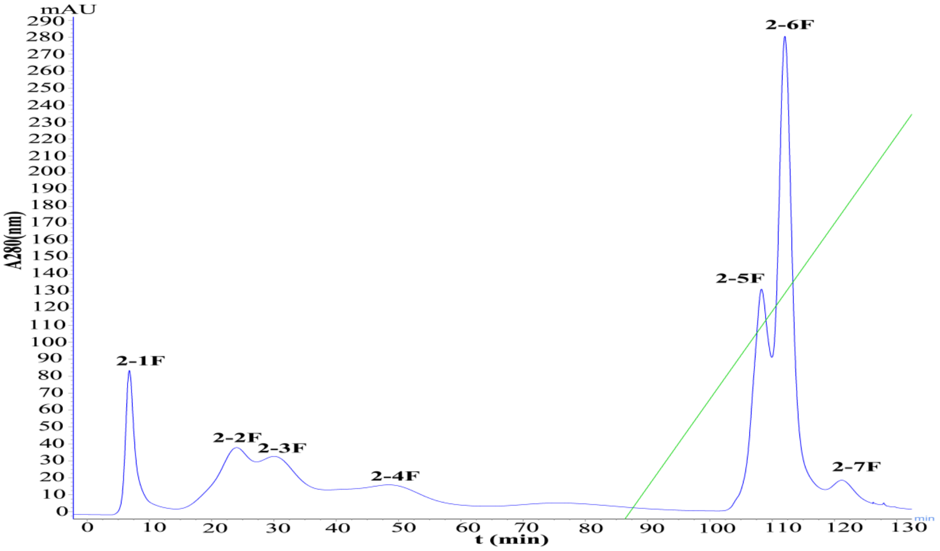

Figure S1 Results of active peak 2F eluted by weak anion exchange chromatography

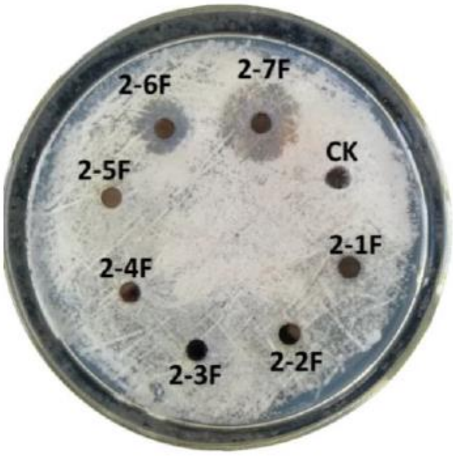

Figure S2 Antifungal effect of elution peak 2-6F (Note: CK for control)

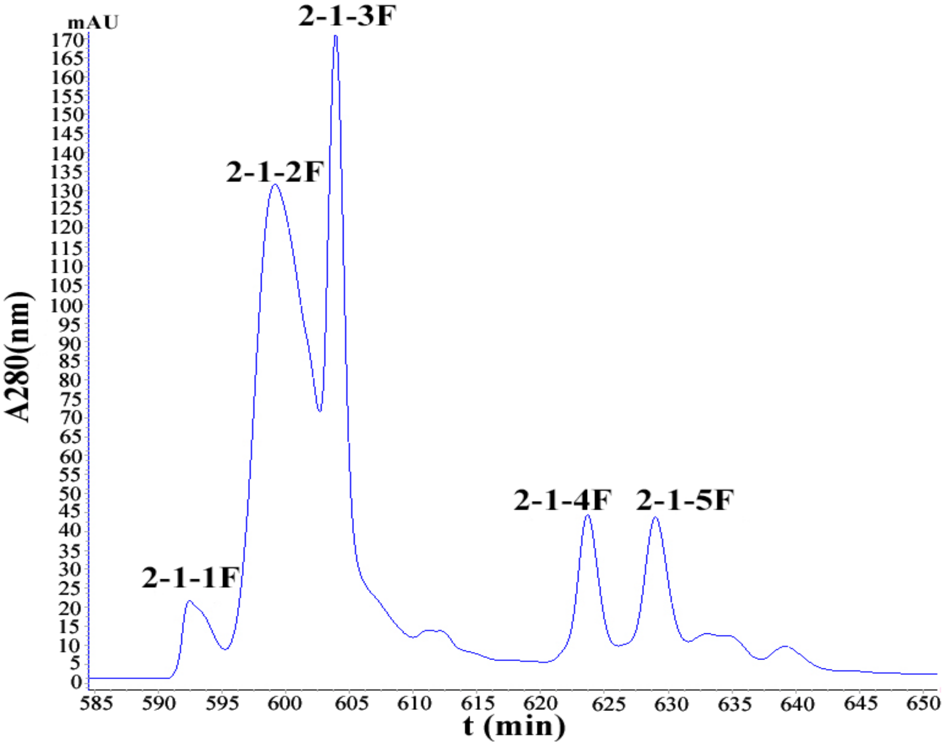

Figure S3 Results of active peak 2-6F eluted by gel filtration chromatography

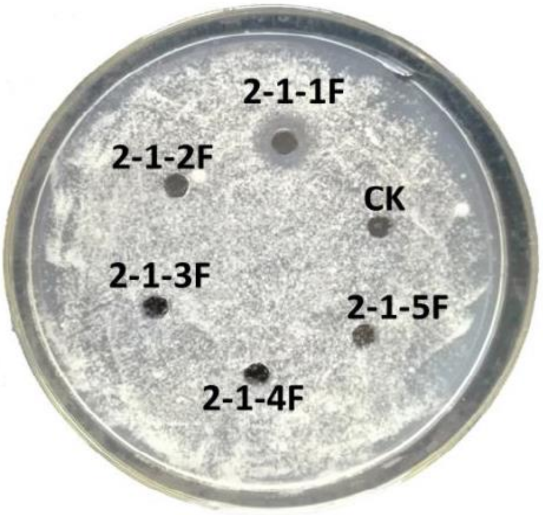

Figure S4 Antifungal effect of elution peak 2-1-1F

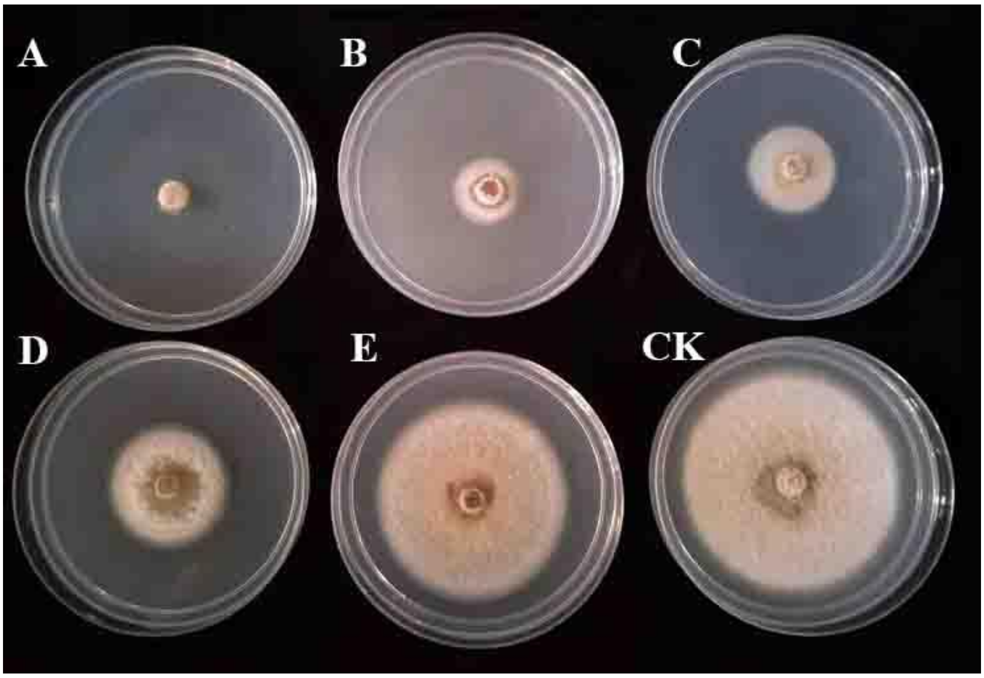

Figure S5 Toxicity test of elution peak 2-1-1F. A) 1.875×10^5 mg/L, B) 1.875×10^4 mg/L, C) 1.875×10^3mg/L, D) 1.875×10^2 mg/L, E) 1.875×10^1 mg/L, CK for control.

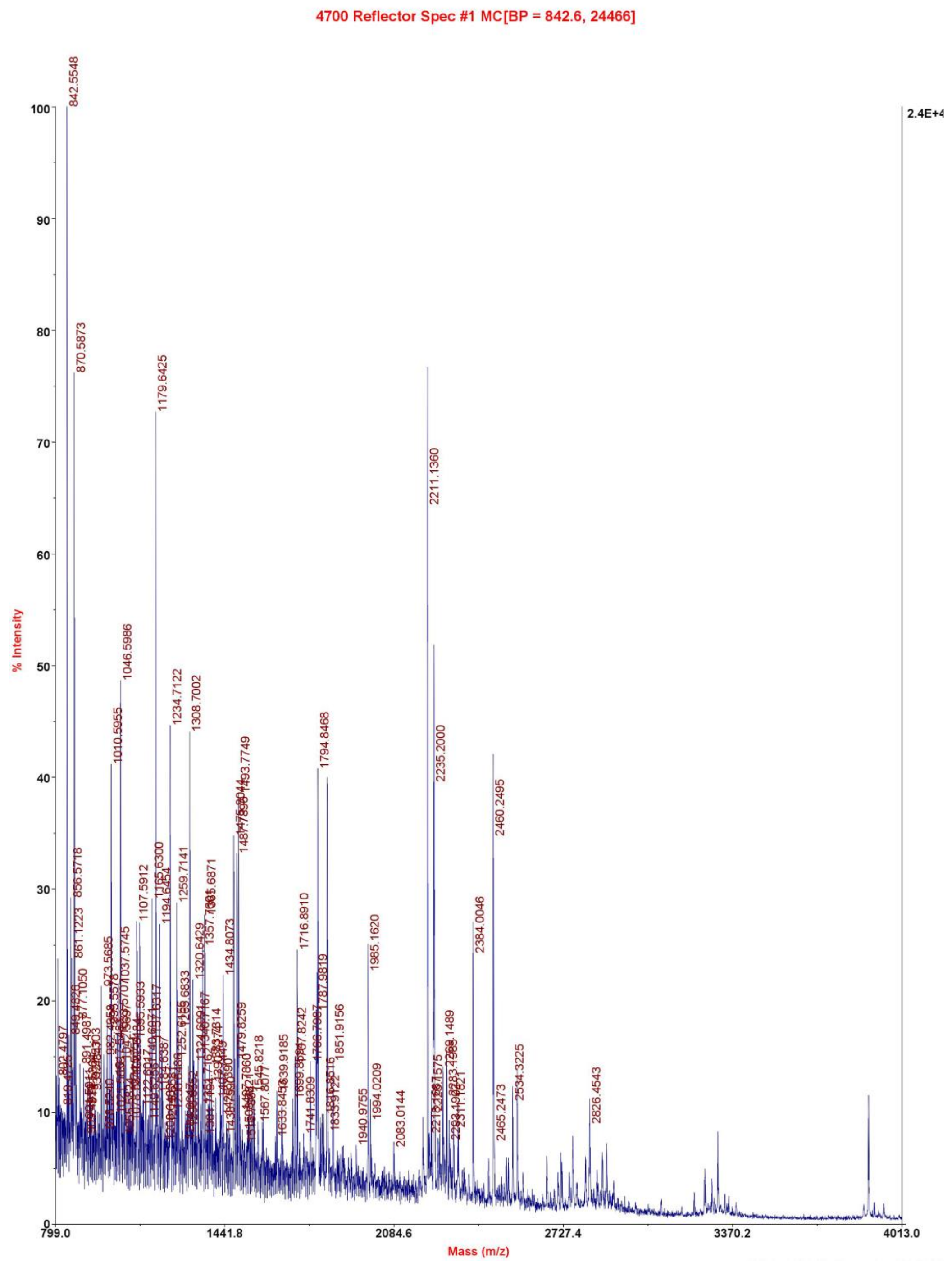

Figure S6 Primary mass spectrum antifungal protein 2-1-1F

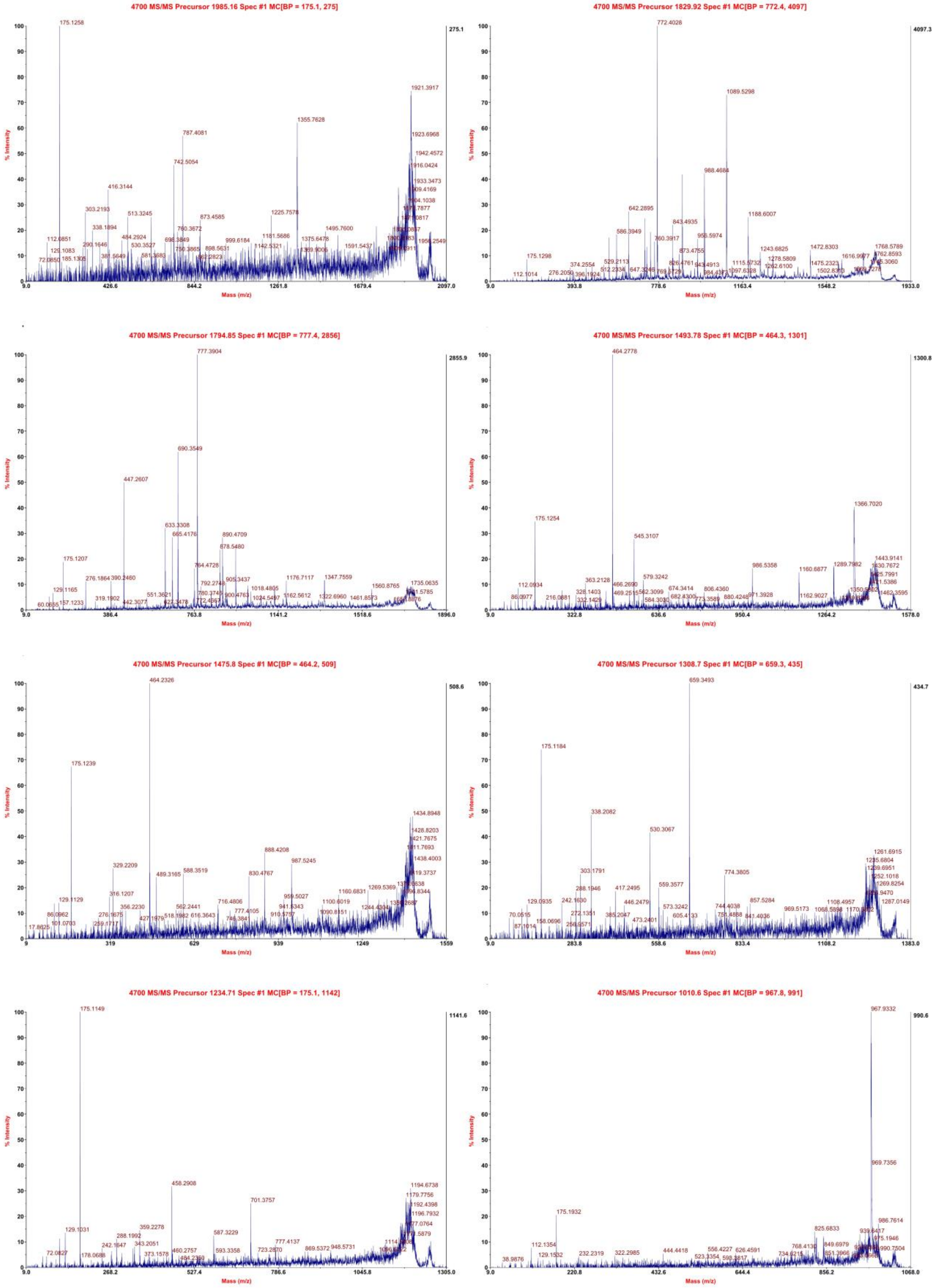

Figure S7 Secondary mass spectrum antifungal protein 2-1-1F

{MATRIX SCIENCE} **Mascot Search Results**

Protein View

Match to: **tr|S6FXJ3|S6FXJ3_9BACI** Score: **394** Expect: **1e-035**
Flagellar hook protein OS=Bacillus velezensis UCMB5033 OX=1338518 GN=flgE PE=3 SV=1

Nominal mass (M_r): **27083**; Calculated pI value: **4.97**
NCBI BLAST search of tr|S6FXJ3|S6FXJ3_9BACI against nr
Unformatted sequence string for pasting into other applications

Variable modifications: Carbamidomethyl (C),Oxidation (M)
Cleavage by Trypsin: cuts C-term side of KR unless next residue is P
Sequence Coverage: **76%**

Matched peptides shown in **Bold Red**

```
  1 MLRSLYSGIS GMKNFQTKLD VIGNNIANVN TVGFKKSRVT FKDMISQTVA
 51 GGSNVTNSKQ IGLGAATSSI DVVHSTGAPQ ATQNKTDLAI DGDGYFQINT
101 GAGIVYTRAG NFGKDNQGNL VTTDGYYLEK IGGGKINIPT DAKDYSIGAD
151 GTVTYTDAGD EVHDAGQIGL VTFPNSAGLE KIGGNLYRES LSSGAASAVT
201 TPGENGTGKL LAGYLEMSNV DLTDEFTEMI VAQRGFQSNS KIITTSDEIL
251 QELVNLKR
```

Figure S8 Mass spectrum identification for antifungal protein 2-1-1F

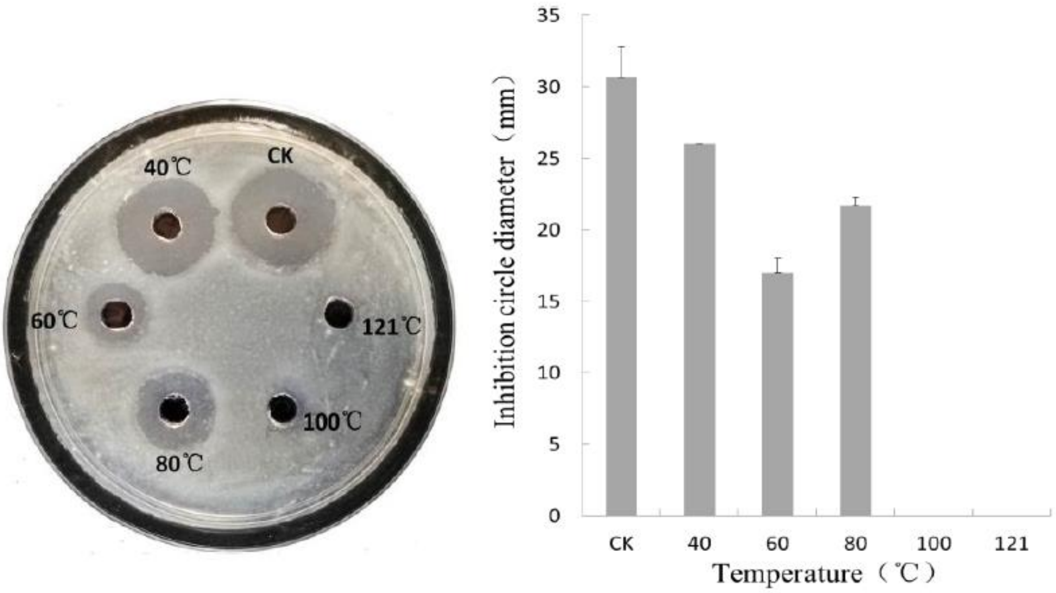

Figure S9 Thermal stability of antifungal crude protein 2F

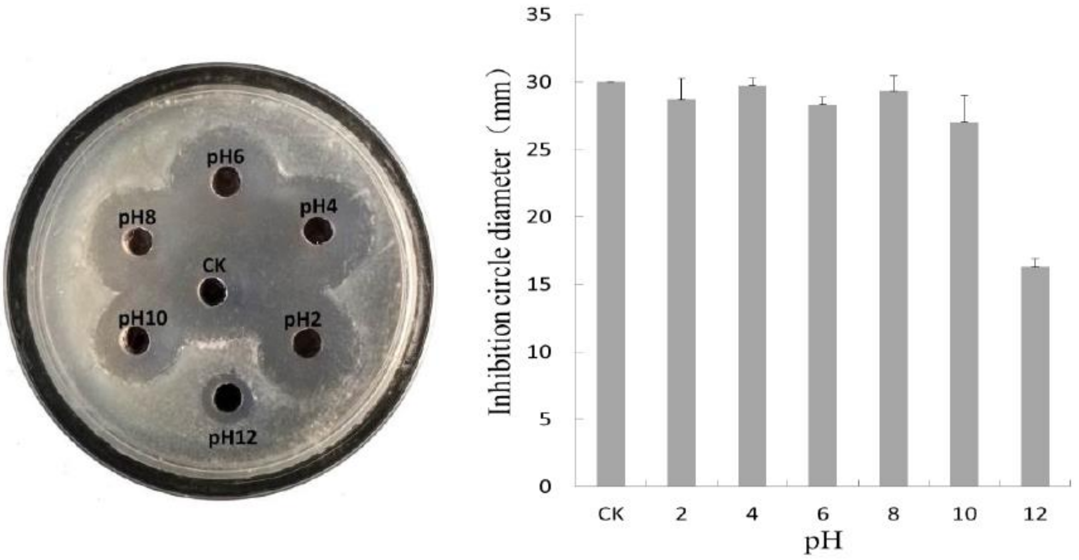

Figure S10 Acid-base stability of antifungal crude protein 2F

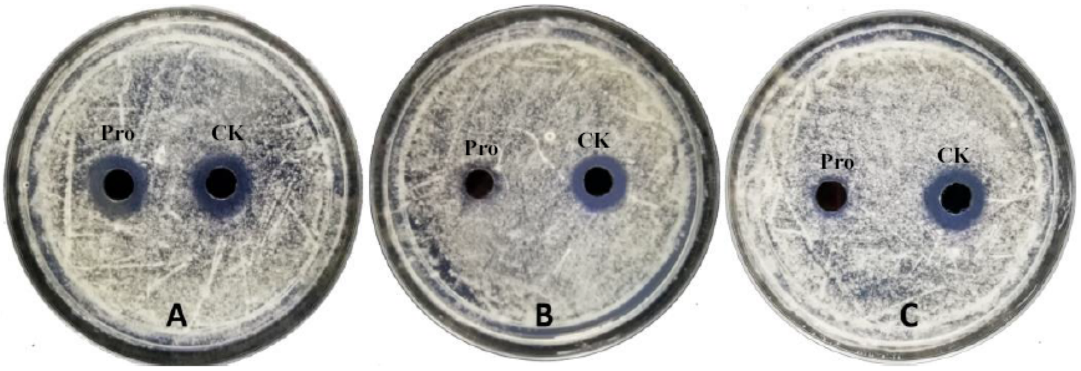

Figure S11 Antifungal crude protein 2F under UV, chloroform, and proteinase K stability (Note: A, B, C for UV, chloroform, proteinase K treatment, respectively)

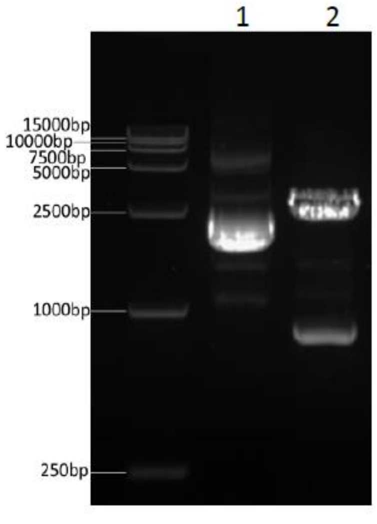

Figure S12 Double enzyme digestion detection of TA cloning vector (Note: 1 for no digestion, 2 for double digestion)

```
Upperline:from1to777 (Clone sequence)
Lowerline:from1to777 (Original sequence)
identity=98.71%(767/777)gap=0.00%(0/777)
1    ATGTTACGTTCATTATATTCCGGTATCAGCGGCATGAAAAACTTTCAGACAAAACTAGAT
1    ATGTTACGTTCATTATATTCCGGTATCAGCGGCATGAAAAACTTTCAGACAAAACTAGAT
61   GTAATCGGCAATAACATCGCCAACGTCAACACAGTGGGATTTAAGAAAAGCCGCGTTACA
61   GTAATCGGCAATAACATCGCCAACGTCAACACAGTGGGATTTAAGAAAAGCCGCGTTACA
121  TTTAAAGATATGATCAGCCAGACAGTCGCCGGCGGTTCAAACGTGACCAATTCAAAACAA
121  TTTAAAGATATGATCAGCCAGACAGTCGCCGGCGGTTCAAACGTGACCAATTCAAAACAA
181  ATCGGCTTAGGTGCCGCGACTTCTTCCATTGATGTTGTTCATTCAACCGGCGCCCCGCAG
181  ATCGGCTTGGGTGCCGCGACTTCTTCCATTGATGTCGTTCATTCAACCGGCGCCCCGCAG
241  GCTACACAGAATAAAACCGACCTAGCCATTGACGGAGACGGCTACTTTCAGATTAATACA
241  GCTACACAGAATAAAACCGACCTAGCCATTGACGGAGACGGATACTTTCAGATTAATACA
301  GGCGCGGGAATTGTTTACACACGTGCGGGGAATTTCGGAAAGGACAATCAAGGGAACCTT
301  GGTTCGGGAATCGTTTACACACGTGCGGGGAATTTCGGAAAGGACAATCAAGGCAACCTT
361  GTTACGACTGACGGGTACTATCTTGAAAAAATCGGCGGCGGCAAAATCAATATTCCGACT
361  GTTACGACTGACGGGTACTACCTTGAAAAAATCGGCGGCGGCAAAATCAATATTCCGACT
421  GATGCGAAAGATTACAGCATCGGTGCAGACGGAACCGTTACATACACTGATGCAGGGGAT
421  GATGCGAAAGATTACAGCATCGGTGCAGACGGAACCGTTACATACACTGATGCAGGGGAT
481  GAGGTTCATGATGCCGGACAAATCGGTCTGGTCACGTTCCCTAACAGCGCCGGTCTGGAA
481  GCGGTTCATGATGCCGGACAAATCGGTCTGGTCACGTTCCCTAACAGCGCCGGTCTGGAA
541  AAAATCGGAGGCAACCTTTACAGAGAATCATTAAGTTCAGGAGCTGCGAGCGCAGTCACC
541  AAAATCGGAGGCAACCTTTATAGAGAATCATTAAGTTCAGGAGCTGCGAGCGCAGTCACC
601  ACTCCGGGCGAAAACGGAACGGGTAAGCTTTTGGCAGGCTATCTTGAAATGTCAAACGTA
601  ACTCCGGGCGAAAACGGAACGGGTAAGCTTTTGGCAGGCTATCTTGAAATGTCAAACGTA
661  GATCTGACAGATGAATTTACCGAAATGATCGTTGCTCAGCGCGGTTTCCAATCAAACTCA
661  GATCTGACAGATGAATTTACCGAAATGATCGTTGCTCAGCGCGGTTTCCAATCAAACTCA
721  AAAATCATTACGACTTCAGATGAAATCCTTCAGGAGCTGGTTAATCTGAAACGGTAA
721  AAAATCATTACGACTTCAGATGAAATCCTTCAGGAGCTGGTTAATCTGAAACGGTAA
```

Figure S13 Result of plasmid sequence of target gene

```
CLUSTAL O(1.2.4) multiple sequence alignment
                                                   Flagellin   family
WP_022553864.1      -------------------MRINHNIAALNTSRQLNAGSNSAAKNMEKLSSGLRINRAGD     41
ABN13608.1          -------------------MRINHNIAALNTSRQLNAGSDSAAKNMEKLSSGLRINRAGD     41
BAB58972.1          -------------------MRINHNIAALNTSRQLNAGSNSAAKNMEKLSSGLRINRAGD     41
WP_015714795.1      -------------------MRINHNIAALNTLNRLSSNNGASQKNMEKLSSGLRINRAGD     41
WP_014665528.1      -------------------MRINHNIAALNTLNRLSSNNGASQKNMEKLSSGLRINRAGD     41
WP_007408329.1      -------------------------------MGMKK-----------KLSLG----VASA     14
                                                       FlgG    family
WP_053602811.1      MLRSLYSGVSGMKNFQTKLDVIGNNIANVGTTGFKKSRVT----FQDTFSQQ----LAGA     52
KIU12641.1          MLRSLYSGISGMKNFQTKLDVIGNNIANVNTVGFKKSRVT----FKDMVSQT----IAGG     52
CDG29557.1          MLRSLYSGISGMKNFQTKLDVIGNNIANVNTVGFKKSRVT----FKDMISQT----VAGG     52
WP_061520525.1      MLRSLYSGISGMKNFQTKLDVIGNNIANVNTVGFKKSRVT----FKDMISQT----VAGG     52
RFU68865.1          MLRSMYSGISGMKNFQTKLDVIGNNIANVNTYGFKKGRVV----FKDLINQD----LTGA     52
WP_053584334.1      MLRSMYSGISGLKNFQTKLDVIGNNIANVNTHGFKKGRVI----FKDLMSQT----QAGA     52


                                                   Flagellin   family
WP_022553864.1      AANDTNTD-SDRSELQKEMDQLSSEV-------TRIS----------------------T     126
ABN13608.1          AANDTNTD-SDRSELQKEMDQLASEV-------TRIS----------------------T     126
BAB58972.1          AANDTNTD-SDRSELQKEMDQLASEV-------TRIS----------------------T     126
WP_015714795.1      ANNTGTQDGTDLGAIQEEIKSLLEEVGGMTGGSKGIS----------------------D     134
WP_014665528.1      AGNTGTQQTEDLTAIKDEMDALTEEI-------DGIS----------------------Q     127
WP_007408329.1      EQS-ANVNLSN---LKPGD-----------------KLTKDFEFRNNGSLAIKEVLMALN     88
                                                       FlgG    family
WP_053602811.1      GE--GYLTVSD------------------EEGETFYSRAGNLYLDQDSRLVTADGLYV--     135
KIU12641.1          GD--GYFRIDT------------------GDG-TAYTREGNFYLDNTGTLVTGDGYHV--     134
CDG29557.1          GD--GYFQINT------------------GAG-IVYTRAGNFGKDNQGNLVTTDGYYL--     128
WP_061520525.1      GD--GYFQINT------------------GSG-IAYTRAGNFGKDNDGNIVTTDGYYL--     128
RFU68865.1          GD--GYFVVGD------------------TAGNESYTRAGNFYLDSTGDLVTGDGLYV--     136
WP_053584334.1      GE--GFFMVAK---YNPNG--DSETIAGESFDNVAYTRAGNFYMDQEGFLVDASGNYV--     148

                                                   Flagellin   family
WP_022553864.1      D-GTLKSGD----GNSTATWADEEV-TDGKVTKEAGYYDDKGALVGSEKLEEGEKLSKGI     231
ABN13608.1          DQNTLTATD----GS-TATWADA----DDATNKPAGYYDAGGKVIASEKLAADSKVTKGI     228
BAB58972.1          DQNTLTSTD----GKSTATWADEVTDPDGKVTKAAGYYDTDGKVIASEKLAADSKVTKGI     233
WP_015714795.1      -----------------TAFKDD-------------------------------------     190
WP_014665528.1      -----------------SA---E-------------------------------------     179
WP_007408329.1      KQDKAAAEA----ISKHIDPK--FLSESGKVNVA--------------------------     171
                                                       FlgG    family
WP_053602811.1      --------------PIPQDATSLTIDEFGTVSYLNN----EN------------QVENAG     170
KIU12641.1          ------------TIKIPTDAQSFSIGSDGKVSIVVA----EG------------KTQDGG     172
CDG29557.1          ------------KINIPTDAKDYSIGADGTVTYTDA----GD------------EVHDAG     166
WP_061520525.1      ------------KINIPTDAKDYSIGPDGNVTYTDS----GD------------QVHNAG     166
RFU68865.1          ----TAGAAFPAIINIPTTATDMSISKNGTITFMDG----GI-------------LRTAG     184
WP_053584334.1      EWEELQTEVVGTRIRIPTDAKSMSIGADGKVTFTDS----EG-------------LKFAG     222


                                                   Flagellin   family
WP_022553864.1      VDMASEM-------MEYTKNNILTQASQAMLAQANQQPQQVLQLLKG-------------     331
ABN13608.1          VDMASEM-------MEYTKNNILTQASQAMLAQANQ------------------------     317
BAB58972.1          VDMASEM-------MEYTKNNILTQASQAMLAQANQQPQQVLQLLKG-------------     333
WP_015714795.1      VDMAKEM-------SEFTKNNILSQASQAMLAQANQQPQNVLQLLR--------------     284
WP_014665528.1      VDMAKEM-------SDFTKNNILSQASQAMLAQANQQPQNVLQLLR--------------     273
WP_007408329.1      KNDTAKTADGLSVQNKFQGNAISLQFSFEATQW------NGLTITKDHTDKDGYVKENEK     254
                                                     FlgG    family
WP_053602811.1      VELSDEFTEMIVGQRGFQANSKIITTSDEILQ--------ELLNLKR-------------     261
KIU12641.1          VDLTDEFTEMIVAQRGFQSNSKIITTSDEILQ--------ELVNLKR-------------     264
CDG29557.1          VDLTDEFTEMIVAQRGFQSNSKIITTSDEILQ--------ELVNLKR-------------     258
WP_061520525.1      VDLTDEFTEMIVAQRGFQSNSKIITTSDEILQ--------ELVNLKR-------------     258
RFU68865.1          VDLSEEFTEMIVAQRAFQSNTRIITTSDEILQ--------ELVNLKR-------------     275
WP_053584334.1      VDLSEEFTEMIVAQRGFQANTRIITTSDEILQ--------ELVNLKR-------------     313
```

Figure S14 Sequence alignment of flagellin conserved regions
